# Supplementary material for: Drug-Eluting, Radiopaque, Tumor-Casting Hydrogels for Endovascular Locoregional Therapy of Hepatocellular Carcinoma
Source: bioRxiv. 2025 Nov 28:2025.11.25.690505. Preprint. [Version 1] doi: 10.1101/2025.11.25.690505 (PMC12703997; doi:10.1101/2025.11.25.690505)
Supplement: Supplement 1 [file media-1.pdf]

Supporting information for:

Drug-Eluting, Radiopaque, Tumor-Casting Hydrogels for Endovascular Locoregional Therapy of Hepatocellular Carcinoma

Yuxi C. Dong,[1,2] Kathleen E. Villaseñor,[1,2] Seokyoung Yoon,[2] Ariful Islam,[2] Luis Vazquez,[2] Alexey Gurevich,[2] Shaun McLaughlin,[2] Terence P. Gade,[1,2]* and David P. Cormode[1,2]*

[1]Department of Bioengineering, University of Pennsylvania, Philadelphia, PA 19104, USA.

[2]Department of Radiology, University of Pennsylvania, 3400 Spruce St, 1 Silverstein, Philadelphia, PA 19104, USA.

* Corresponding author

E-mail: tgade@pennmedicine.upenn.edu, david.cormode@pennmedicine.upenn.edu

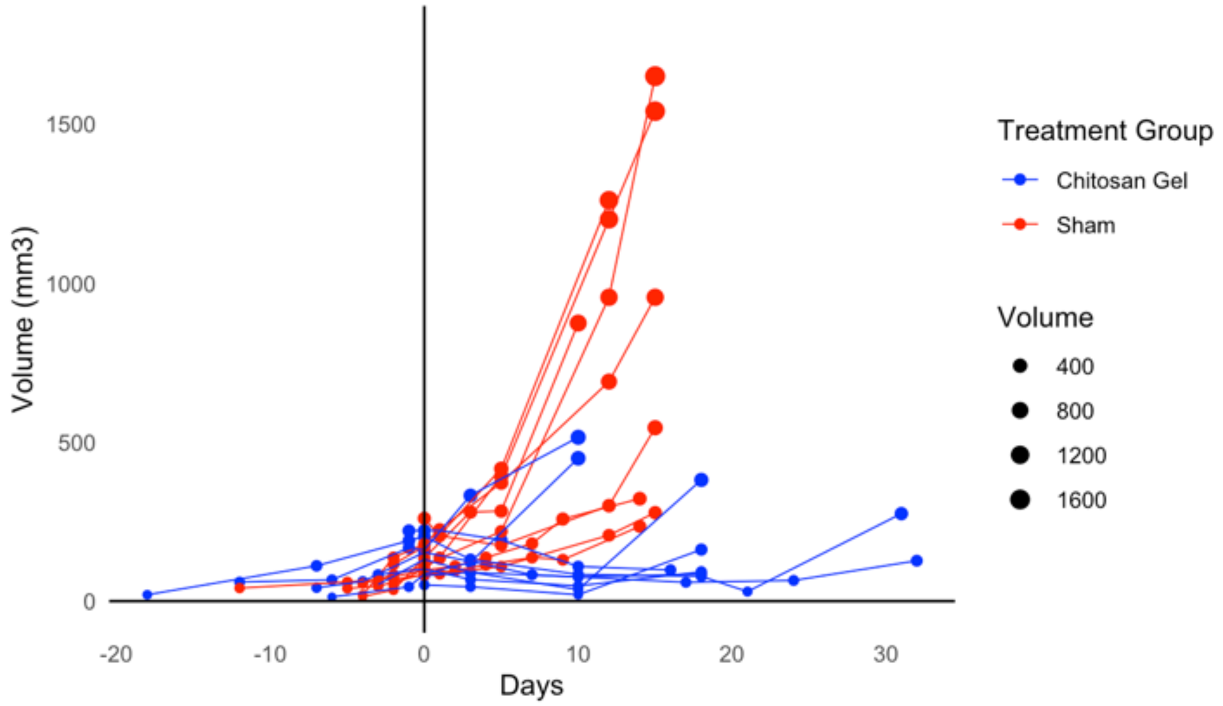

Figure S1. Comparison of the growth curves of tumors treated either with sham or chitosan gel.

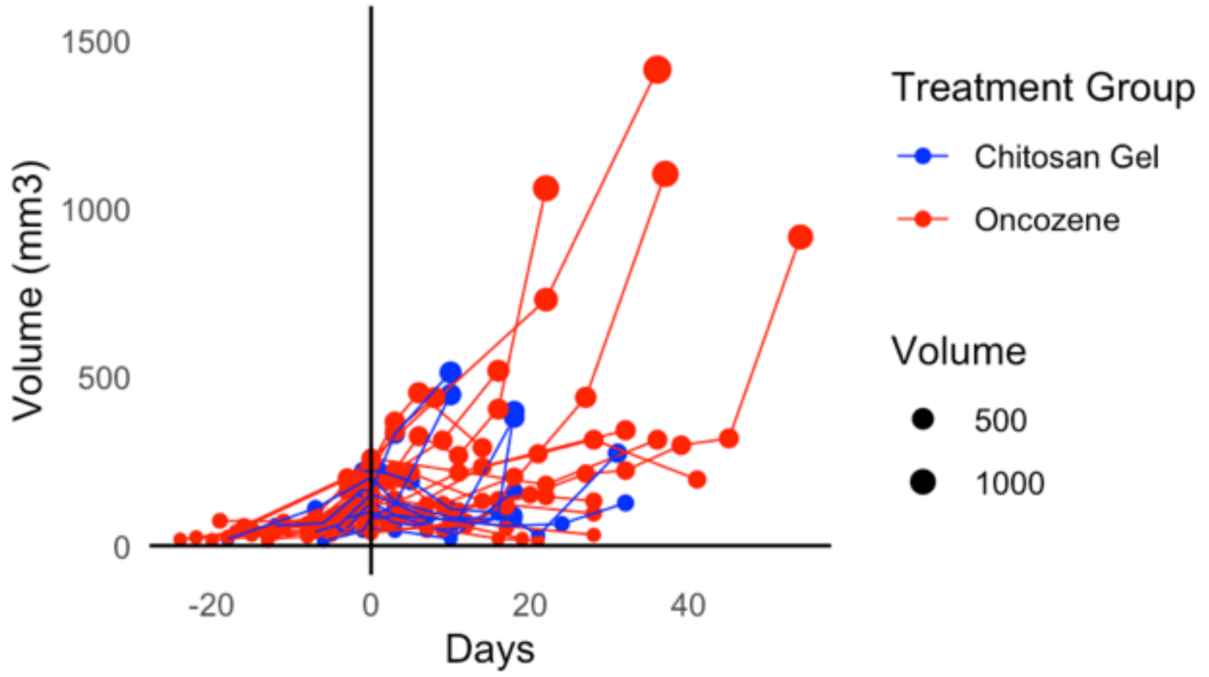

Figure S2. Comparison of the growth curves of tumors treated either with Oncozene beads or chitosan gel.
